# Thank you to Reviewers 2020

**DOI:** 10.1002/cam4.3758

**Published:** 2021-02-11

Thank you to Reviewers 2020

The Editor‐in‐Chief and the Editorial team gratefully acknowledge the below individuals who served as peer reviewers for Cancer Medicine during 2020.

Aas, Kirsti

Abbaci, Muriel

Abd Elnaser, Anwar

Abdelhakiem, Mohamed

Abdollahpour‐Alitappeh, Meghdad

Abdou, Yara

Abdullah, Khatijah Lim

Abenavoli, Ludovico

Abraham, Devaprabu

Abraham, Philip

Abruzzi, Amy

Abufaraj, Mohammad

Accordino, Melissa

Acharya, Srijan

Adam, JF

Adam, Salome

Adambekov, Shalkar

Adams, Brian

Adams, Hugo J. A.

Adamy, Guy

Adedeji, Isaac

Adzika, Gabriel

Agarwal, Charu

Agarwal, Shailesh

Aghdam, Nima

Agrawal, Surbhi

Ahangari, G.

Ahmed, Nuzhat

Ahmed, Sairah

Ahn, Dongbin

Akahane, Koshi

Akimoto, Tetsuo

Aksoy, Asude

Alaeddini, Mojgan

Alaniz, Laura

Albano, Domenico

Alese, Olatunji

Alexander, Sarah

AlFaar, Ahmad

Algee‐Hewitt, Bridget F.B.

Ali, Askal A

Alip, Adlinda

Allegra, Eugenia

Allodji, Rodrigue

Alongi, Filippo

Alphandery, Edouard

Al‐Refaie, Waddah

Altomonte, Jennifer

Al‐Toubah, Taymeyah

Alvarez‐Fernandez, Sheila Maria

Amaral Mendes, Rui

Amundadottir, Laufey

An, Sanqi

An, Thuy

An, Yi

Anand, Shashi

Anastasov, Natasa

Anderson, Benjamin

Ando, Koji

Andre, Nicolas

Andre, Thierry

Angajala, Anusha

Annunziata, Salvatore

Anttila, Ahti

Anuar, Nur Adila

Aparicio, Thomas

Apperley, Jane

Aragon‐Ching, Jeanny

Arai, Gaku

Arandjelovic, Ognjen

Araujo, Daniel

Archer, Stephanie

Arita, Hideyuki

Armitage, James

Arora, M.

Arun, Banu

Ashley, Natalie

Athale, Uma

Atuegwu, Nkiruka

Au, Qingyan

Augestad, Knut M

August, David

Aung, Winn

Autier, Philippe

Awasthi, Shivanshu

Awick, Elizabeth A.

Ay, Cihan

Aypar, Eda

Azar, Ibrahim

Baba, Hideo

Baccarani, Michele

Back, Michael

Badami, Sunil

Badr, Gamal

Badrising, Sushil

Baech, Joachim

Baetz, Tara

Bagge, Roger Olofsson

Bagheri, Nader

Bahrabadi, Mehrdad

Bahrami, Naeim

Bai, Rui

Bai, Xue

Bainbridge, Matthew

Bakhshandeh, Haleh

Bakhti , Seyedeh Zahra

Bakouny, Ziad

Ballas, Leslie

Balleari, Enrico

Ballester, Leomar Y.

Baloch, Zulqarnain

Banerjee, Sreeparna

Bange, Erin

Bao, Heling

Bao, Lei

Bao, Li

Baracos, Vicki

Barata, Pedro

Barnes, PJ

Barnhart, Robert

Barr, Ronald

Barrera Herrera, Luis

Barros, Bruna

Barta, Stefan

Bartlett, Nancy L.

Basch, Ethan

Bashir, Mohamad

Basho, Reva

Bassan, Renato

Basu, Partha

Basulto Martinez, Mario

Battaglia, Yuri

Batura, Deepak

Bauer, Alexandria

Bazan, Jose

Beckman, Robert

Beharee, Nitish

Beirne, James

Belibasakis, Georgios

Bello, Ibrahim

Benderra, Marc‐Antoine

Bennett, John

Benusiglio, Patrick

Berdeja, Jesus

Berghen, Charlien

Berglund, Anders

Bernard, Stephen

Berry, Jesse L.

Bertolo, Riccardo

Besson, Caroline

Besutti, Giulia

Betof, Allison

Bhat, Ambarish P.

Bhat, Ganapati

Bhatia, Smita

Bhatia, Prateek

Bhattacharjee, Anukana

Bian, Shelly X.

Bian, Yun

Bian, Zehua

Bien, Ewa

Bilen, Mehmet A

Biliatis, Ioannis

Binder, Adam

Biondi, Andrea

Bishop, Karen

Bisoffi, Marco

Bjarnason, Georg

Blackburn, Anneke C.

Blaes, Anne

Blomqvist, Carl

Boakye, Dany

Boeri, Mattia

Boichuk, Sergei

Boldrini, Luca

Bonde, Jesper

Bonifacio, Massimiliano

Bonin, Serena

Boot, Annemieke

Booth, Neill

Borazanci, Erkut

Bosisio, Francesca

Botter, Sander M.

Botticella, Angela

Bottomley, Matthew

Bou‐Dargham, Mayassa

Bouffet, Eric

Bourlon, Maria

Bouvet, Michael

Braga, Sonia

Branford, Susan

Brasil, Sergio

Brassil, Kelly

Breccia, Massimo

Brenner, Annette K.

Brest, Patrick

Brock, Katharine

Broer, Stefan

Brohl, Andrew

Brotkin, Samuel

Brown, Kevin

Bryant, Curtis

Buchbinder, Elizabeth

Buckstein, Rena

Bumma, Naresh

Bunch, Brittany

Burbos, Nikolaos

Burgess, Andrew

Burillo‐Sanz, Sergio

Burnham, Leanne

Cabot, Myles

Caetano, Oliveira R

Cagney, Daniel

Cai, Gengming

Cai, Guoxiang

Cai, Ren

Cai, Sanjun

Cai, Xianlei

Cai, Zhi

Calvez‐Kelm, Florence Le

Campacci, Natalia

Campi, Riccardo

Campos, Nicole

Canzian, Federico

Cao, Dedong

Cao, Lei

Cao, Qizhi

Cao, Wei

Cao, XinPing

Capelozzi, Vera

Capocaccia, Riccardo

Caraglia, Michele

Carleton, Neil

Carlino, Matteo S.

Carpen, Timo

Carr, Robert

Carrasco Pro, Sebastian

Cash, Hannes

Castro, Anna

Cates, Justin

Cayota, Alfonso

Ceccarelli, Simona

Celio, Luigi

Cella, David

Cerchione, C

Chadburn, Amy

Chaddad, Ahmad

Chai, Ke‐qun

Chamuleau, Martin

Chan, Aden K.

Chan, Dedrick

Chan, Jimmy Yu‐Wai

Chandramohan, Anuradha

Chang, Hui

Chang, I‐Wei

Chang, Jinjia

Chang, Wenjun

Chantada, Guillermo

Chao, Changjiang

Chapman, Christina

Chatelut, Etienne

Chatzopoulos, Kyriakos

Chaudhari, Rishabh

Chauhan, Nar Singh

Chavez, Julio

Che, Xiaofang

Checcucci, Enrico

Check, Devon

Chen, Yongsong

Chen, Bobin

Chen, Chen

Chen, Chia‐Yu

Chen, Chien‐Chih

Chen, Chuanben

Chen, Dung‐Tsa

Chen, Fang

Chen, Gang

Chen, Hongda

Chen, Jia‐jian

Chen, Jianfeng

Chen, Jian‐Guo

Chen, Jiapeng

Chen, Jie

Chen, Jui‐Chieh

Chen, Jun

Chen, Junqiang

Chen, Laing

Chen, Lei

Chen, Lizhang

Chen, Luke

Chen, Ming

Chen, Qing

Chen, Runsheng

Chen, Shih‐Hsiang

Chen, Tianxiang

Chen, Wanqing

Chen, Wei

Chen, Xi

Chen, Xiaonan

Chen, Xiaosong

Chen, Xingdong

Chen, Xing‐xing

Chen, Xu

Chen, Yan

Chen, Yang‐Chao

Chen, Yijiao

Chen, Yinting

Chen, Yongbing

Chen, Yongshun

Chen, Yuhan

Chen, Zhen

Chen, Zhishui

Chen, Zhong

Cheng, Lei

Cheng, Liang

Cheng, Qing

Cheng, Qiu‐Yan

Cheng, Quan

Cheng, Shiyuan

Cheng, Ying

Cheng, Yingsheng

Cheng, Yufeng

Cheng, Yung‐Jen

Cherian, Anish Jacob

Cheung, Chi Yuen

Chhabra, Saurabh

Chiang, Veronica

Chien, Chih‐Yen

Chien, Huei‐Tzu

Chiesa Estomba, Carlos

Chmielowski, Bartosz

Ch'ng, Ewe Seng

Cho, Hyo Jung

Cho, Je‐Yoel

Cho, William C. S.

Cho, Yong Beom

Choi, Seungtaek

Chong, Choon Seng

Chou, Wen‐Chi

Chouaid, Christos

Chouvardas, Panagiotis

Chow, Daniel S.

Chow, Edward

Chow, Ronald

Christiani, David

Chu, Minjie

Chua, Melvin

Chuang, Po‐Heng

Chunyan, Du

Chutipongtanate, Somchai

Cianchetti, Marco

Cimadamore, Alessia

Clary, Alecia

Clifford, Gary

Cockerell, Clay

Coles, Theresa

Collins, Sean P.

Combes, J.D

Comenzo, Raymond L.

Comont, Thibault

Cong, Xianling

Contel, Maria

Cook, Michael B.

Cooney, Kathleen

Copp, Tessa

Cornish, Alex

Corrias, Maria V Corrias

Cortellini, Alessio

Costanzo, Erin

Cox, Kim

Craig, Andrew W.

Cranmer, Lee

Crispen, Paul

Cubiella, Joaquin

Cuccia, Francesco

Cuesta‐Guardiola, Tatiana

Cui, Jie

Cui, Jiuwei

Cui, Juan

Cui, Kaisa

Cui, Rongjun

Cui, Shuxia

Cui, Yuanbo

Cui, Zhaolei

Cullen, Jennifer

Cwynarski, Kate

Cybulski, Marek

Dai, Chenyang

Dai, Dong Jun

Dai, Juncheng

Dai, Ruiwu

Dai, Shuang

Dai, Wei‐Jie

Dai, Xiangpeng

Dai, Xiaofeng

Dai, Yu‐Jun

Dai, Zhijun

Dai, Zhi‐Jun

Dal Maso, Luigino

Dalla Via, Jack

Damia, Giovanna

Damiani, Daniela

Damodar, Sharat

Dandan, Xiong

Dann, Eldad

Davar, Diwakar

Davidoff, Amy

Davidson, Brittany

De Bernardi, Elisabetta

de Biase, Dario

de Blank, Peter

de Bock, Geertruida

De Giorgi, Ugo

De Moor, Janet

de Smith, Adam

De Vincentis, Giuseppe

de Vries, Esther

Dean, Michael

Decker, Thomas

Deek, M.

Defraene, Gilles

Degboe, Arnold

Denburg, Avram

Deng, Dao‐Xing

Deng, Sisi

Deng, Wei

Deng, Youping

Devarakonda, Srinivas

Dey, Biswajit

Di Carlo, Veronica

Di Ieva, Antonio

Di Lorenzo, Giuseppe

di Pietro, Massimiliano

Diamantopoulos, Panagiotis

Dickson, Paxton

Dimartino, Lisa

Dimitrova, Nadya

Ding, Kefeng

Ding, Qianshan

Ding, Ruoxi

Ding, Xiangya

Ding, Yi

Ding, Yongbin

Ding, Yongfeng

DiPippo, Vincent A.

Dirven, Linda

Dittmar, T.

Djordjevic, Bojana

Długosz‐Danecka, Monika

Dodd, Natalie

Dogan, Haluk

Domínguez‐Vigil, Irma Guadalupe

Dong, Di

Dong, Jing

Dong, Sheng

Dong, Ting

Dong, Zhiqiang

Dotan, Efrat

Du, Changzheng

Du, Mulong

Du, Mulong

Du, Shasha

Dubashi, Biswajit

Dubrovin, Vasilii

Dueck, Amylou

Dumitrascu, Traian

Durocher, Francine

Dustler, Magnus

Dutta, Pranab

Dutta, Shubham

Dutta, Soumitra

Dvorak, Pavel

Dyer, Martin J.S.

Dziedzic, Robert

Ebrahimabadi, Maryam

Ediz, Caner

Effat Panah, Hosein

Ehrbar, Verena

Ehrhardt, Mathew

Ehrstedt, Christoffer

Eigentler, Thomas

Eisenmann, Eric

El Ghoch, Marwan

El Osta, Badi

El Sayed, Mohamed

Elattar, Ahmed

Elias, Anthony

Eloranta, Sandra

Elsamadicy, Aladine

Emadi, Ashkan

Engelhardt, Monika

Engholm, Gerda

Enikeev, Dmitry

Epperla, Narendranath

Erber, Wendy

Eskazan, Ahmet Emre

Essani, Karim

Esteva, Magdalena

Etwebi, Zienab

Eun, Jung Woo

Evans, James J.

Fabbrini, P

Fabian, Flora

Facoetti, Angelica

Fallara, Giuseppe

Falzone, Luca

Fan, Daidi

Fan, Jia

Fan, Jinhai

Fan, Lihua

Fan, Ruitai

Fan, Shaonan

Fan, Xiaoming

Fan, Xinjian

Fan, Zhimin

Fang, Bingliang

Fang, Chuantao

Fang, Li‐Wen

Fawzy, Manal

Fawzy, Manal S.

Feinberg, Mark

Feng, Chenchen

Feng, Lei

Feng, Ninghan

Feng, Qianxi

Feng, Qinfu

Feng, Wang

Fernandes, Monica

Ferrari, Andrea

Ferro, Matteo

Ferrucci, Pier Francesco

Finch, Emily

Finlay, David

Finucane, Anne M.

Fioresi, Rita

Fiori, Cristian

Foldi, Julia

Fountzilas, Christos

Fradet, Vincent

Francis, Steve

Francisci, Silvia

Francolini, Giulio

Freire, Guilherme

Freitas, Leandro

Frountzas, Maximos

Fu, Bin

Fu, Caiyun

Fu, Denggang

Fu, Guanghou

Fu, Junjiang

Fu, Kai

Fu, Liyun

Fu, WeiHua

Fu, Zhenming

Fujii, Takeo

Fujii, Yasuhisa

Fujita, Kohei

Fujiwara, Yutaka

Fukuda, Minoru

Fukumura, Yuki

Fukunaga, Yosuke

Fukuokaya , W.

Furness, Kate

Furuta, Junichi

Furuta, Saori

Fuse, Kyoko

Fushida, Sachio

Gaillet, Bruno

Gainetdinov, Ildar V.

Gainor, Justin

Gale, Robert Peter

Gallamini, Andrea

Galmiche, Antoine

Gan, Gin

Gandhi, Ajeet

Gandia Aguado, David

Ganesan, Prasanth

Ganta, Ranga R.

Gao, Hui

Gao, Peng

Gao, Shaowei

Gao, Xian Hua

Gao, Yadong

Gao, Yi‐Bo

Gao, Yong

Gao, Yuan

Gao, Yuen

Gao‐pei, Zhu

Garcia‐Gutierrez, Valentin

Garg, Tullika

Gariboldi, Manuela

Garnier, Louis

Gascon, Pere

Gaspard, Nicholas

Gatt, Moshe

Gautheret, Daniel

Ge, Weiting

Geerts, Dirk

Geier, Margaux

Geng, Hua

Gennaro, Nicolo

George, Biju

George, Gincy

George, Prema

Geyer, Julia T

Ghanbari, Reza

Ghanbarian, Hossein

Gharahkhani, Puya Gharahkhani

Ghavami, Saeid

Ghidini, Michele

Ghorbani, Saied

Ghosh‐Laskar, Sarbani

Giaj Levra, Niccolò

Gibson, Michael

Gibson, Paul

Gill, Sharlene

Giordano, Ricardo J.

Giraldi, Laura

Gjermundson, Hali J.

Glimelius, Ingrid

Glover, Anthony

Gobin, Romila

Gobitti, Carlo

Goel, R.

Goemans, Bianca

Goldstein, Benjamin

Goldvaser, Hadar

Gong, Chang

Gong, Jianfeng

Gong, Jian‐Ping

Gong, Li

Gong, Yan

Gonzalez Castro, Luis

González‐San Segundo, Carmen

Goodwin, Rory

Goto, Hiroaki

Gou, Xin

Gower, Adam

Greenup, Rachel

Greipp, Patricia

Grignani, Giovanni

Grivas, Petros

Grossmann, Kenneth

Grotz, Travis

Grummet, Jeremy P

Grzegrzolka, Jedrzej

Gu, Jian

Gu, Jun

Gu, Ke

Gu, Yayun

Gu, Yuanyuan

Guan, Hongya

Guan, Xiaoxiang

Gui, Benedetta

Gungormez, Cigdem

Gunn, Christine

Guo, An‐Yuan

Guo, Boda

Guo, Feng

Guo, Jiwei

Guo, Jun

Guo, Lanwei

Guo, Shicheng

Guo, Yuming

Gupta, Abha A.

Gupta, Amit

Gupta, Ritu

Gupta, Sudeep

Haas, Oscar

Haferlach, Torsten

Hagiwara, Sumitaka

Haider, Stefan

Ham, Won Sik

Hamady, Zaed ZR

Hambly, Brett

Han, Cheng‐Bo

Han, Fei

Han, Yong

Han, Yuyan

Han, Zhijun

Hanafy, Nemany

Hang, Jun‐jie

Hannah‐Shmouni, Fady

Hannum, Susan

Hanprasertpong, Jitti

Hans, Didier

Hao, Pan

Harada, Toshiyuki

Harano, Kenichi

Harding, James

Hardy, Kristina

Harky, Amer

Harraz, A.

Harris, Andrew

Harris, Anne

Harrison, Claire

Hartman, Douglas

Hartman, William

Hashimoto, Kohei

Hashimoto, Takeshi

Hassan, Bass

Hassan, Dastsooz

Hassel, Jessica

Hata, Masaharu

Hatakeyama, Shingo

Hatefi, Arash

Haveman, Lianne

Hawk, Ernest

Hayashi, Hideyuki

Hayashi, Kazuhiko

Hayden, Patrick

He, Jing

He, Kang

He, Miao

He, Qifei

He, Weiliang

He, Wen‐zhuo

He, Yijing

He, Yiping

He, Yongfeng

He, Yuting

Heckman, Caroline

Hefermehl, Lukas

Hegde, John

Hektoen, Helga

Helgason, Vignir

Helland, Åslaug

Henson, Christina

Hepner, Adriana

Hertler, Andrew

Hess, Moritz

Hibbert, Peter

Hildesheim, Allan

Hill, Brian

Himoto, Takashi

Hiratsuka, Yusuke

Hirayama, Alexandre

Hird, Amanda

Ho, Yuh‐Shan

Hoffmeister, Michael

Homma, Akihiro

Hong, Dongwan

Hong, Peng

Hong, Weiguo

Hong, Young‐Rock

Honma, Yoshitaka

Hoonakker, Peter

Horii, Rika

Horimoto, Yoshiya

Horinouchi, Hidehito

Horstmann, Martin A.

Horta, Martinho‐Campolina‐Rebello

Hou, Baohua

Hou, Hsin‐An

Hou, Ming‐Feng

Hou, Yi‐Chou

Houri, Hamid Reza

Hsiao, Chao‐Pin

Hsiao, Kuei‐Yang

Hsieh, Jason Chia‐Hsun

Hsieh, Ming‐Han

Hsieh, Po‐Fan

Hsu, Hui‐Ping

Hsu, Yi‐Chiung

Hsueh, Shun‐Wen

Hu, Chaosu

Hu, Daixing

Hu, Hai‐jie

Hu, Jian‐Kun

Hu, Jifan

Hu, Junjie

Hu, Minggen

Hu, Silong

Hu, Wangxiong

Hu, Xuelian

Hu, Zheng

Hu, Zhicheng

Hua, Chung‐Ching

Hua, Yunpeng

Huang, Chang‐Ming

Huang, Chia‐Yen

Huang, Dan

Huang, Gloria

Huang, Hai

Huang, Hai‐Neng

Huang, Huai‐Hsuan

Huang, Jianfei

Huang, Junjie

Huang, Lu

Huang, Mingtao

Huang, Peng

Huang, Shenglin

Huang, Tao

Huang, Weidong

Huang, Yen‐Ming

Huang, Yongye

Huang, Yuehua

Huang, Yuhan

Huang, Zhangheng

Huang, Zhaohui

Huang, Zongqiang

Hui, David

Huntington, Scott

Huppert, Laura

Hwang, Ming‐Jing

Hyland, Kelly

Iacovelli, Roberto

Ide, Hiroki

Ihemelandu, Chukwuemeka

Ilic, Irena

Imai, Reiko

Imaizumi, Kazuyoshi

Imamura, Toshihiko

Ingrosso, Gianluca

Inigo, Joseph

Intaraphet, Suthida

Inyushin, Mikhail

Ip, Andrew

Ippolito, Davide

Irrinki, Krishna

Irtan, Sabine

Isenberg‐Grzeda, Elie

Ito, Katsuhiro

Ito, Yasuhiro

Itoh, Shinji

Iyer, Prajish

Jagannath, Sundar

Jakob, Jens

Jalali, Pooya

James, Francis

Janda, Monika

Jang, Soong‐Nang

Janvilisri, Tavan

Jarrard, David F.

Jaspers, J P M

Javadinia, Seyed Alireza

Jayapalan, Jaime

Jeltsch, Albert

Jeng, Wen‐Juei

Jensen, Lars Henrik

Jeong, Chang Wook

Jeong, Keun‐Yeong

Jereczek, Barbara

Jerkeman, Mats

Ji, Jiansong

Jia, Ruipeng

Jia, Weihua

Jiang, Chuanlu

Jiang, Dewei

Jiang, Guozhong

Jiang, Mingzuo

Jiang, Ning

Jiang, Sunfang

Jiang, Tongchao

Jiang, Xiaoyin

Jiang, Xingming

Jiang, Yafen

Jiang, Yi

Jiang, Yuming

Jiao, Wenjie

Jiao, Yan

Jiao, Zheng

Jie, Junqin

Jim, Heather

Jin, Feng

Jin, Guangfu

Jin, Kairui

Jin, Kideok

Jin, Weina

Jin, Xin

Jin, Xun

Jin, Zhaohui

Jo, Young Suk

Joeres, Fabian

Johanning, Gary

John, Joseph

Johnson, J.

Johnston, Donna

Johnston, Emily

Johnston, Robert

Johnstone, Peter

Jones, Robin

Jones, Joshua

Jose, Wesley

Joshi, Bharat

Joshi, Shreyas

Jou, Janice

Joung, Je‐Gun

ju, shaoqing

Jun, He

Junck, Larry

Jung, Kwang Hwa

Juonala, Markus

Jurczak, Wojciech

Kaehler, Katharina C.

Kahn, Ben

Kaisaki, Pamela J.

Kaito, Akio

Kakeji, Yoshihiro

Kale, Hrishikesh

Kamrava, Mitchell

Kanai, Osamu

Kanda, Tatsuo

Kanemitsu, Yukihide

Kanesvaran, Ravindran

Kang, Hyunseok

Kang, Jennifer

Kang, Xiaozheng

Kang, Yoontae

Kann, Benjamin

Kansy, Benjamin A.

Kantidakis, Theo

Kapoor, Prabodh

Karanth, Siddharth

Karia, Pritesh

Karim, Safiya

Karimi, Seyed M.

Karpathiou, Georgia

Karpiński, Tomasz

Karube, Kennosuke

Karunakaran, Udayakumar

Kashiwagi, Shinichiro

Kather, J.

Kato, Shingo

Katsui, Kuniaki

Katsuta, Eriko

Kattan, Michael

Kaufman, Jonathan

Kauppila, Joonas

Kaur, Kulvinder

Kaushik, Nagendra Kumar

Kawakita, Mutsushi

Kawaoka, Tomokazu

Kayal, Smita

Kazemi, Tohid

Ke, Ai‐wu

Ke, Chongwei

Ke, Zhi‐Bin

Keating, Nancy

Keck, Tobias

Kehl, Kenneth

Kelgiorgi, Dionysia

Keller, Gisela

Kemper, Marius

Kensler, Kevin H.

Kenzik, Kelly M.

Kern, Peter

Khalid, Omar

Khamar, Bakulesh

Khan, Aziz

Khan, Imran

Khan, Mohammad Aslam

Khan, Naazneen

Khattry, Naveen

Kibbe, Warren

Kikawa, Yuichiro

Kim, Yeul‐Hong

Kim, Dong Chul

Kim, Hyoung‐Il

Kim, Jeongseon

Kim, Jin Hyoung

Kim, Kun Hyung

Kim, Sang‐We

Kim, Se Hyun

Kim, Sinyoung

Kim, Sung Soo

Kim, Tae Hyun

Kim, Tae Min

Kim, Yun Hak

King, Martin T.

Kitamura, Katsuya

Klein, Jens

Kline, Cassie

Klinkhammer‐Schalke, Monika

Knappskog, Stian

Kobayashi, Takashi

Kobayashi, Tsuyoshi

Kochuparambi, Samith Thomas

Koëter, T

Koga, Fumitaka

Koga, Yoshikatsu

Kogawa, Takahiro

Kohno, Takashi

Koie, Takuya

Kojima, Yasushi

Kok, Victor

koksal, hakan

Komatsu, Norio

Kommalapati, Anuhya

Kondo, Satoru

Kondo, Takeshi

Kone, Moumouni

Kong, Rui

Kos, Spela

Koshiol, Jill

Kosugi, S.

Kotoula, Vassiliki

Kourelis, Taxiarchis

Kowalski, Luiz Paulo

Krishnatreya, Manigreeva

Krishnatry, Rahul

Kruser, Timothy

Krušlin, Božo

Kubota, Kaoru

Kuittinen, Outi

Kumar, Abhijeet

Kumar, Lalit

Kumar, Shaji K.

Kumar, Sushil

Kume, Haruki

Künzel, Julian

Kuo, Sung‐Hsin

Kuppen, Peter

Kuriyama, A.

Kuru, Bekir

Kusec, Rajko

Kuzuya, Teiji

Kwok, Young

Kyriazoglou, Anastasios

La Forgia, Daniele

La Vecchia, Carlo

Labaki, Chris

Labbe, Catherine

Lachowie, Curtis

Laetsch, Theodore

Laios, Alexandros

Lan, Huiyin

Lan, Qing

Lan, Yu

Landau, Heather

Landwehr, Michelle

Lao, Xiang‐Ming

Lauren, Fontana

Lawlor, Peter

Lawson Morris, David

Lazzari, Chiara

Lazzi, Stefano

Leach, Brandi

Lee, Anna

Lee, Bae Hoon

Lee, Cho‐Rok

Lee, Han Hong

Lee, Kyu Sang

Lee, Kyuwan

Lee, Tsung‐Hsien

Lee, Ying‐Ray

Legge, Francesco

Lehrer, Eric

Lei, Jun

Lei, Zhe

Leithner, Andreas

Leleu, Xavier

Lertbutsayanukul, Chawalit

Leshno, Moshe

Lester, Susan

Li, Chuzhong

Li, Xiaoxing

Li, A‐bing

Li, Bingkun

Li, Cai

Li, Chong

Li, Chunying

Li, Dongmei

Li, Fang

Li, Fu‐yu

Li, Fuzhou

Li, Guoxin

Li, Haoran

Li, Hongyu

Li, Hua

Li, Jian

Li, Jianbin

Li, Jiancheng

Li, Jianping

Li, Jin

Li, Jing

Li, Jinglong

Li, Jinluan

Li, Jinyun

Li, Juan

Li, lanjuan

Li, Le‐Qun

Li, Lifeng

Li, Lingchao

Li, Lun

Li, Mengyuan

Li, Min

Li, Mingshan

Li, Na

Li, Pan

Li, Qiaochuan

Li, Qiao‐Qiao

Li, Qiu

Li, Qiyuan

Li, Rongkun

Li, Rutian

Li, Sin‐Syue

Li, Tong

Li, Tuan‐Jie

Li, Wang

Li, Wei

Li, Xiao

Li, Xiaoman

Li, Xingrui

Li, Xinrong

Li, Xuejun

Li, Yan

Li, Yiming

Li, Yongning

Li, Yuan

Li, Yuan

Li, Yu‐hong

Li, Zhanzhan

Li, Zhen

Li, Zhi

Li, Zhi‐Cheng

Li, Zhuang

Liang, Geyu

Liang, Jiaqi

Liang, Jie‐ying

Liao, Rui

Liao, Weijia

Liao, Xi‐Wen

Libra, Massimo

Lieberman, Paul M.

Lim, Jasmine

Lin, Fan

Lin, Ge

Lin, Hao‐Yu

Lin, Jianxian

Lin, Peng

Lin, Peter P.

Lin, Qin

Lin, Wei Yu

Lin, Yao

Lipitz‐Snyderman, Allison

Little, Julian

Liu, Bo

Liu, Chang

Liu, Changan

Liu, Chia‐Jen

Liu, Dai

Liu, Feng

Liu, Gao

Liu, Hailang

Liu, Hao

Liu, Huanliang

Liu, Jie

Liu, Jinsong

Liu, Kai

Liu, Kuangzheng

Liu, Lianxin

Liu, Lihua

Liu, Lingxiang

Liu, Lipin

Liu, Minetta

Liu, Mingming

Liu, Panpan

Liu, Ping

Liu, Qiang

Liu, Qifa

Liu, Rong

Liu, Songbai

Liu, Tianshu

Liu, Wanli

Liu, Wei

Liu, Wei‐Hsiu

Liu, Xiaofeng

Liu, Xiaoguang

Liu, Xiaoping

Liu, Xiaowen

Liu, Xinpeng

Liu, Xinru

Liu, Xuejun

Liu, Yan

Liu, Yang

Liu, Yong

Liu, Yuanbo

Liu, Zeyi

Liu, Zhi‐Jie

Liu, Zhiqiang

Liu, Zhiwei

Liu, Zuqiang

Lobo, João

Locatello, Luca

Loi, Michele

London, Wendy

Long, Haixia

Long, Pan

Lopez, Giselle

Lopez‐Yrigoyen, Martha

Lorrey, Selena

Lu, Shuxiong

Lu, Donghao

Lu, Jiachun

Lu, Jiade

Lu, Jinjian

Lu, Lina

Lu, Qingchun

Lu, Renquan

Lu, Tianzhu

Lu, TP

Lu, Weiqin

Lu, Weiyue

Lu, Xiaojie

Lu, Xin

Lucas Jr., John

Luginbuhl, Adam

Lukkahatai, Nada

Lukong, Kiven Erique

Luminari, Stefano

Luo, Danmeng

Luo, Dixian

Luo, Lin

Luo, Minglei

Luo, Minhua

Luo, Shiwen

Luo, Shuhong

Lupichuk, Sasha

Lupo, Philip

Lussana, Federico

Luu, Hung

Luzzago, Stefano

Lv, Yi

Lv, Yufeng

Lyall, Amanda E.

Lyu, Jun

Ma, Changchun

Ma, Chaoya

Ma, Cynthia

Ma, Hongxia

Ma, Jun

Ma, Nianhan

Ma, Rong

Ma, Vincent

Ma, Xiaoxin

Ma, Zhongliang

Mackay, Tara

Macrae, Finlay

Maeda, Osamu

Mahabir, Somdat

Mai, Hai‐Qiang

Makarem, Maisam

Malapelle, Umberto

Malhotra, Hemant

Malumbres, Marcos

Mandati, Vinay

Mandeville, Henry

Mangoni, Arduina

Manne, Ashish

Mao, JunJ.

Mao, Yingying

Marano, Luigi

Marasco, Giovanni

Maraveyas, Anthony

Marion‐Gallois, Roland

Marrone, M.

Martin, Michelle

Martinelli, Giovanni

Martin‐Orozco, Elena

Maschio, Marta

Mascitti, Marco

Massari, Francesco

Masuko, Takashi

Matboli, Marwa

Materin, Miguel

Mather, Karen

Mathevet, Patrice

Mato, Anthony

Matsuda, Kazuyuki

Matsuo, Keitaro

Matsuo, Yukinori

Matsuzaki, Junko

Matthews, Kevin

Maughan, Benjamin

Mauri, Giovanni

Mavridis, Konstantinos

Mayah, Wael Wahid

Mayer, Deborah K

Mazzola, Rosario

McCoy, Matthew

McFarland, Daniel Curtis

Mcormack, David

Mego, M.

Mehdipour, Fareshteh

Mehta‐Shah, Neha

Mei, Zhechuan

Mei, Zubing

Melchior, Nicole

Melin, Beatrice

Mendel, Ehud

Menendez, Carolyn

Meng, Jialin

Meng, Xiang‐Yu

Mercieca‐Bebber, Rebecca

Merks, Johannes H. M.

Mesa, Ruben A.

Metzner, Bernd

Meyer, Alberto

Meyers, Travis

Mian, Omar

Miao, Lei

Miao, Xiaoping

Miaskowski, Christine

Middeldorp, Jaap

Midorikawa, Yutaka

Mihara, Keichiro

Miller, Anthony

Min, Chang‐Ki

Mina, Roberto

Mircea, Tampa

Mirmiran, Parvin

Mistro, Annarosa Del

Mitiushkina, Natalia V

Mitsunaga, Shuichi

Miura, Satoru

Miyazawa, Yoshiyuki

Miyoshi, Hiroaki

Mizukami, Takuro

Mnich, Katarzyna

Mo, Chengqiang

Mo, Qianxing

Mo, Shaobo

Mo, Xiaofei

Moineddin, Rahim

Mojarad, Ehsan

Momose, Isao

Monga, Varun

Montazeri, Ali

Moon, Mi Hyoung

Mooradian, Meghan

Moore, Elizabeth

Morandi, Luca

Mortezaee, Keywan

Moser, Justin

Mota, Jose Mauricio

Mousli, A.

Mowery, Yvonne

Muhuri, Manish

Mukhtar, Fahad

Murphy, Neil

Murthy, Guru

Mussai, Francis

Mwansisya, Tumbwene

Na, Niu

Nabel, Christopher

Nagane, Motoo

Nagaraju, Ganji

Nagasaka, Kazunori

Nagler, Arnon

Nair, Sreenath

Nair, Velu

Nakachi, Kohei

Nakagawa, Hidetoshi

Nakagawa, Tohru

Nakajima, Koichiro

Nakalembe, Miriam

Nakano, Masahito

Nakayama, Yujiro

Nan, Hongmei

Nanyingi, Miisa

Natkunam, Yasodha

Navai, Shoba

Navarria, Pierina

Nawaz, Muhammad

Naya, Yukio

Nazha, Bassel

Nedrow, Jessie

Negri, Francesca

Neil, Jordan Matthew

Neupane, Prakash

Neupane, Subas

Neuwelt, Edward

Ng, C. K. Y.

Ng, Lui

Ng, Siok Bian

Ng, Sweet Ping

Nguyen, Dung Quang

Ni, Shujuan

Nichols, Anthony C.

Nicksic, Nicole E.

Niclou, Simone P.

Nicosia, Luca

Nie, Yongzhan

Niedzielski, Joshua S.

Nieto, Yago

Nikiforov, Yuri E.

Nikolaenko, L

Ning, Gang

Ning, Pengbo

Nio, Koki

Niranjan, Soumya

Nishino, Ryuhei

Nishio, Shin

Niu, Jixiang

Nodeh, Mohammad

Noji, Takehiro

Noma, Kazuhiro

Nooka, Ajay

Novince, Chad

Nunès, Jacques

Nylander, Karin

Nylén, Carolina

Obeng‐Gyasi, Samilia

Ochs‐Balcom, Heather M.

Offner, Fritz

Ogawa, Asao

Oh, Chang‐Mo

O'Hara, James

Ohashi, Akihiro

Ohashi, Manabu

Ohtsubo, Koushiro

Ohtsuki, Sumio

Okada, Keiko

Okamoto, Hiroshi

Okamoto, Yasuo

Okazuka, Kiyoshi

Oki, Eiji

Oki, Yasuhiro

Okines, Alicia

Okuma, Yusuke

Okuyama, Hiroyuki

Olmetto, Emanuela

O'Loughlin, Declan

Olsen, Catherine

Olszewski, Adam

Omae, Kenji

Onal, Cem

Onozawa, Mizuki

Ordi, Jaume

O'Reilly, Lorraine A.

Orgel, Etan

Orlandi, Ester

Orsey, Andrea

Ostrom, Quinn

Ou, Rongying

Ouyang, Yan

Owen, Patrick

Özgüzer, Alp

Ozpinar, Aysel

Ozretić, Petar

Pabinger, Ingrid

Paltiel, Ora

Palumbo, Giuseppe

Pamanes, Claudia

Pan, Hen‐Chih

Pan, Jay

Pan, Jing‐Hua

Pan, Shang‐Ling

Pan, Xuejun

Panchal, Hina

Pandit, Harshul

Pang, Tao

Panzuto, Francesco

Papayannidis, Cristina

Parada, Humberto

Parayath, Neha

Parikh, Nainesh

Park, Haeseong

Park, Henry

Park, Jeong‐Yeol

Park, Jong

Park, Sung‐Gyoo

Parys, Jan B.

Paskett, Electra

Pastore, Antonio Luigi

Pataer, Apar

Patil, Nirav

Patton, Mary

Pavithran, K

Payer, Juraj

Peeters, Marc

Pekmezci, Melike

Peng, Junsheng

Peng, Kai‐Lin

Peng, Sui

Peng, Tao

Peng, Yihan

Peng, Zhen‐Wei

Peng, Zhiyu

Pereira, Aloysius

Perez, Kimberly

Perez‐Cornago, Aurora

Pesce, Caterina

Peters, Anthea

Petrikaite, Vilma

Petrova, Dafina

Pezzella, Francesco

Pezzolo, Annalisa

Phelan, Rachel

Philpot, Lindsey

Picchio, Maria

Piccinato, Carla de Azevedo

Piccirillo, Rosanna

Picozzi, Vincent

Pietroletti, Renato

Pignot, Geraldine

Pine, Sharon

Pinheiro, Paulo S.

Pinkawa, Michael

Pinzi, Valentina

Pirtoli, Luigi

Pisapia, Pasquale

Pitts, Todd M.

Piva, Terrence J.

Planck, Maria

Polesel, Jerry

Poluzzi, Elisabetta

Pombo‐de‐Oliveira, Maria S.

Poplawski, Tomasz

Popper, Helmut

Portillo, Isabel

Pouliakis, Abraham

Pourquier, Philippe

Powell, Arfon

Pradere, Benjamin

Prasad, Birbal

Pratap, Uday

Prebet, Thomas

Prieto, Ruth

Prieto, Victor

Prizment, Anna

Probst, Pascal

Prosperi, Jenifer R.

Prpić, Marin

Prsic, Elizabeth

Pruthi, Ankur

Pu, Zening

Punyadeera, Chamindie

Purohit, Rituraj

Qazilbash, Mohd

Qi, Feng

Qi, Xiaowei

Qian, Chao‐nan (Miles)

Qian, Feng

Qian, Jianfei

Qian, Jin

Qian, Wen‐Bin

Qian, Xiaohua

Qian, Xiaojun

Qiao, Fengchang

Qin, Guoyou

Qin, Na

Qin, Yanmei

Qin, Ya‐Zhen

Qiu, Hai‐Bo

Qiu, Hong

Qiu, Jiangdong

Qiu, Lihua

Qiu, Mantang

Qiu, Su

Qiu, Sufang

Qiu, Ying

Qiu, Zeting

Qu, Bin

Qu, Jinrong

Qu, Xiujuan

Quaini, Federico

Qureshi, Amran

R, van Hillegersberg

R. I., Anu

Rachek, Lyudmila

Radhakrishnan, Venkatraman

Radhakrishnan, Vivek

Radocha, Jakub

Raftery, Daniel

Rahib, Lola

Rahouma, Mohamed

Raj, Nitya

Raj, Stalin

Rajagopalan, Anugraha

Ramaiah, M. Janaki

Ramakrishnan, Seshadri

Ramani, Vinod

Rambaldi, Alessandro

Ramirez de Molina, Ana

Ramirez, Julia

Ramos‐Esquivel, Allan

Ramos‐Garcia, Pablo

Ranganathan, Balu

Rangarajan, R.

Rangarajan, Venkatesh

Rao, Devika

Rao, Ragunatha

Raphael, Jacques

Rathod, Sanjay

Rau, Prof. Horst

Ravandi, Farhad

Reagan, John

Recher, Christian

Recondo, Gonzalo

Redell, Michelle

Reeve, Bryce

Reilly, Marie

Reimer, Toralf

Reiner, Anne

Ren, Jun

Ren, Ning

Ren, Shengxiang

Ren, XiangHai

Ren, Xuehan

Ren, Zefang

Rezaee, Seyyed Abdolrahim

Ribeiro, Karina

Riedel, Richard F.

Rizzari, Carmelo

Robijns, Jolien

Robinson, David

Robsahm, Trude

Rodgers, Linda

Rödiger, Stefan

Rodriguez‐Dorantes, Mauricio

Rohrmann, Sabine

Rombi, Barbara

Romero, Sally

Roque, Dana

Roser, Katharina

Rosti, Gianantonio

Roth, Michael

Rotte, Anand

Routh, Jonathan

Rovito, Michael

Roy Chowdhuri, Sinchita

Ruan, Hailong

Ruan, Shanming

Ruiz‐Cordero, Roberto

Russell, Larihu

Russo, Antonio

Russo, Suzanne

Rustgi, Sheila

Rustin, G

Rusu, Laura‐Cristina

Rutter, Carolyn M.

Saab, Raya

Saba, Nabil

Sabol, Maja

Sad, Lamiss

Sadik, Ahmed

Saeki, Issei

Saglio, Giuseppe

Sahoo, Debashis

Sakai, Ken

Sakamoto, Shinichi

Sakaue, Tomohisa

Salles, Gilles

Salmasi, Amirali

Salo, Tuula

Samadani, Ali Akbar

Sanabria, Alvaro

Sanderson, Maureen

Sang, Shaowei

San‐Julian, Mikel

Sannigrahi, Malay

Sarian, Luis Otavio

Sarrias, Maria‐Rosa

Sasaki, Mitsuhito

Sasayama, Takashi

Sato, Kazuhiko

Sato, Masaya

Sato, Yuichi

Sauer, Christopher

Saylor, Philip J.

Scarpi, Emanuela

Schaeffeler, Norbert

Schafer, Eric

Scherer, Dominique

Schips, Luigi

Schlaepfer, IR

Schlechter, Ben

Schmaier, Alvin

Schmidt, Heike

Schmitt, Fernando

Schneider, Barbara

Schraw, Jeremy

Schueler, Julia

Schwartzberg, Lee

Scoazec, Jean‐Yves

Seishima, Jun

Seiter, Karen

Seitz, Guido

Seki, Akihiro

Semmes, Eleanor

Sengar, Manju

Seth, Tulika

Sethi, Gautam

Sewell, William

Seymour, John

Shah, Manish

Shahjaman, MD

Shaikh, Mushfiq

Shair, Kathy H. Y.

Shaish, Hiram

Shalata, Walid

Shamseddine, Ali

Shan, Jingxuan

Shang, Changzhen

Shang, Jun

Shang, Song

Shang, Xiaoling

Shao, Xianhao

Shao, Yang

Sharma, Amod

Sharma, Sumit

Shen, Chan

Shen, Erica

Shen, Feng

Shen, Fu

Shen, Jianfei

Shen, Qiang

Shen, Shunli

Shen, Xian

Shen, Yan

Shen, Yanyun

Sheng, Weiqi

Sher, Farooq

Sherafatian, Masih

Shi, Xianquan

Shi, Hanping

Shi, Jianxiang

Shi, Ke‐Qing

Shi, Keqing

Shi, Tao

Shi, Xiao‐ming

Shi, Yuankai

Shiba, Satoshi

Shiddiky, Muhammad

Shigeoka, Manabu

Shih, Helen

Shillington, Alicia

Shim, Ju Hyun

Shima, Kosuke

Shimada, Hideaki

Shimada, Kazuyuki

Shimizu, Satoshi

Shimoi, Tatsunori

Shin, Dong Wook

Shinomiya, Nariyoshi

Shiota, Masaki

Short, Nicholas

Shroff, Rachna

Shukla, Sudhanshu Kumar

Si, Lu

Si, Yiran

Sica, Antonello

Siddharth, Sumit

Siddique, Abu Bakar

Siddique, Hifzur

Sidi, Hatta

Sidiqi, Hasib

Siebers, Albert

Sier, Cornelis

Silva, Amélia M.

Silva, Andrew

Silvestris, Nicola

Singh, Parminder

Singh, Pawan

Singh, Ratnakar

Singh, Shiv

Siok‐Fong, Chin

Siriwardhana, Chathura

Slager, Susan

Smith, Megan Alexandra

Smith, Robert

Soh, Junichi

Sokolov, I.

Solanki, Abhishek

Solinas, Cinzia

Song, Bin

Song, Ci

Song, Fangzhou

Song, Jukun

Song, Qian

Song, Xiaofeng

Song, Yongxi

Song, Zhengshuai

Song, Zhihui

Sorbye, Halfdan

Søreide, Kjetil

Sosman, Jeffrey

Souza, Priscila de

Spicer, Timothy

Spigelman, Allan

Sridhar, Srikala

Srivastava, Lalit Mohan

Staaf, Johan

Stathopoulos, Georgios

Steinberg, Julia

Stelljes, Matthias

Steuer, Conor

Stiggelbout, Anne M.

Stolnicu, Simona

Straus, David

Strayhorn, Shaila

Stricker, Thomas

Strickland, Stephen

Strobbe, L. J. A.

Stupp, Roger

Su, Chien‐Wei

Su, Chunxia

Su, Hao

Sudan, Sarabjeet

Suh, Sang‐Yeon

Sui, Xinbing

Sulaiman, Siti Aishah

Sullivan, Christopher

Sultan, Iyad

Sun, Cheng‐Cao

Sun, James

Sun, JianSong

Sun, Leitao

Sun, Qiao‐Yang

Sun, Shi‐Yong

Sun, Xiaochun

Sun, Xin

Sun, Xueying

Sun, Yang

Sun, Ying

Sun, Yuping

Sunagozaka, Hajime

Sunakawa, Yu

Sundling, Kaitlin

Suneja, Gita

Sung, Wen‐Wei

Surbatovic, Maja

Sureda, Anna

Surov, Alexey

Suzuki, Masataka

Suzuki, Miho

Suzuki, Rei

Suzuki, Ritsuro

Syahir, Amir

Szlosarek, P. W.

Taarnhøj, Gry

Tadi, Surender

Tadmor, Tamar

Tagliabue, Marta

Taha, Diaa Eldin

Tai, Elizabeth

Taigo, Kato

Takagi, Toshio

Takahashi, Hidenori

Takami, Akiyoshi

Takano, Shigetsugu

Takayama, Kenichi

Takefumi, Komiya

Takeuchi, Kengo

Talamantes, Sarah

Tamiya, Akihiro

Tamiya, Motohiro

Tan, Aik Choon

Tan, Li

Tan, Mingjia

Tan, Weige

Tanabe, Katsuyuki

Tanaka, Shinji

Tanaka, Yasuhito

Tang, Dong

Tang, Guilin

Tang, Hailin

Tang, Jiajia

Tang, Min

Tang, Nanhong

Tang, Xiaowen

Tangjitgamol, Siriwan

Tanner, Lynn

Tanuma, Nobuhiro

Tao, Huishan

Tao, Wensi

Tao, Yi

Tavassoly, Iman

Tayel, Safaa

Teer, Jamie

Teimoori‐Toolabi, Ladan

Teleka, Stanley

Teng, Lisong

Teow, Sin‐Yeang

Thakral, Beenu

Thakur, Nisha

Thall, Peter F.

Thein, Hla‐Hla

Thomas, Teresa

Thong, Melissa

Thoudam, Themis

Threatt, Stevie

Tian, Junqiang

Tian, Suyan

Tian, Yantao

Tian, Yaping

Tian, Yuan

Tian, Zelin

Tiao, Gregory

Ting, David T.

Tiribelli, Mario

Tobberup, Randi

Tokuhira, Michihide

Tomita, Naoto

Tomizawa, Daisuke

Tomuleasa, Ciprian

Torres‐Saavedra, Pedro

Tosar, Juan Pablo

Toshima, Fumihito

Tovoli, Francesco

Townsend, Jeff

Toya, Takumi

Tracy, Kirsten

Treps, Lucas

Trevisi, Gianluca

Triggiani, Luca

Trimble, Edward

Trippa, Fabio

Troiano, Giuseppe

Troncone, Giancarlo

Tsang, Derek

Tschudin, Sibil

Tse, Gary M.

Tsikouras, Panagiotis

Tsoukalas, Nikos

Tsuchiya, Kaoru

Tsuji, Akihito

Tsuji, Kunihiro

Tsuzuki, Toyonori

Tu, JianCheng

Tupikowski, Krzysztof

Turic, Bojana

Udupa, Karthik

Uetake, Hiroyuki

Uhlig, Annemarie

Ujiie, Hideki

Ulivi, Paola

Undas, A

Unger, Elizabeth R

Upadhyay, Arun

Urabe, Masayuki

V, Lei X

Vaishampayan, Ulka

Vale, Diama

Valverde, Claudia

Valverde, Patricia

van der Geest, Lydia

Van Hemelrijck, Mieke

Van Mater, David

Van Swearingen, Amanda

van Tol‐Geerdink, J. J.

VandenBussche, Christopher J.

Vaux, David

Väyrynen, Juha

Vázquez‐Cornejo, Edmundo

Veeraiah, Surendran

Vegnenegre, Alain

Velmurugan, Bharath Kumar

Verma, Avanti

Verma, Nitin

Vermunt, Marit

Verschraegen, Claire

Vicente, Juan C. de

Vicini, Frank

Vikas, Praveen

Villani, Anita

Villarreal, Cynthia

Vincenzi, Bruno

Virgo, Katherine S.

Visco, Carlo

Visser, Otto

Viswanath, Satish

Viswas, Raja Solomon

Vogl, Dan

von Itzstein, Mitchell

von Mehren, Margaret

von Neuhoff, Nils

Vortmeyer, Alexander

Vosberg, Sebastian

Voutsadakis, Ioannis A

Vranic, Semir

Vreeland, Timothy

Sébastien Wälchli

Wadhwa, Aman

Wages, Nolan

Wagner, Lars

Wakatsuki, Masaru

Wakimoto, Hiroaki

Wan, Fengyi

Wan, Yinsheng

Wang, Wei

Wang, Aifen

Wang, Chendong

Wang, Cheng

Wang, Chih Yuan

Wang, Chongkai

Wang, Chuanxin

Wang, Fahui

Wang, Fangzheng

Wang, Guoying

Wang, Haiyong

Wang, Hong

Wang, Hongzhi

Wang, Hua

Wang, Hung‐Wei

Wang, Jaw‐Yuan

Wang, Jianbing

Wang, Jianming

Wang, Jiazhou

Wang, Jijia

Wang, Jingpu

Wang, Ju

Wang, Jun

Wang, Kefeng

Wang, Kewei

Wang, Kun

Wang, L.

Wang, Le

Wang, Ming

Wang, Peihua

Wang, Peng‐Hui

Wang, Qi

Wang, Qian

Wang, Qianghu

Wang, Qilong

Wang, Renjie

Wang, Rong

Wang, ShaoMing

Wang, Shixuan

Wang, Shouhan

Wang, Shuyuan

Wang, Wen‐Lun

Wang, Xiao Fei

Wang, Xiao‐Bing

Wang, Xiaochen

Wang, Xicheng

Wang, Xinjun

Wang, Xinqiu

Wang, Xiongjun

Wang, Xue

Wang, Xuejuan

Wang, Yanqing

Wang, Yanqiu

Wang, Yihong

Wang, Yiqin

Wang, Yixiang

Wang, Yong

Wang, Yongzhi

Wang, Youxin

Wang, Yuexiang

Wang, Yugang

Wang, Yu‐Ming

Wang, Yuzhuo

Wang, Zhao

Wang, Zhenning

Wang, Zhichao

Wang, Zhixian

Wang, Ziheng

Wang, Zi‐Xian

Warman, Matthew

Washington, Samuel

Wassersug, Richard

Watanabe, Junichiro

Watanabe, Kazuo

Weaver, Sallie

Webb, Myfanwy

Wee, Joseph

Wei, Kang

Wei, Jia

Wei, Jianhua

Wei, Jinli

Wei, Sheng

Wei, Ye

Wei, Yongyue

Wei, Zheng

Weigl, Matthias

Weipu, Mao

Weiss, Jared

Welsh, James

Wen, Qing‐Lian

WenJun, Cheng

Werner, Theresa

Wernli, Karen J.

Wertz, Jasmin

Wessely, Anja

Whelan, Jeremy

Whiteside, Theresa

Wietrzyk, Joanna

Wight, Joel

Wilhite, Annelise

Wilkins, Simon

Willard, Victoria

Williams, Courtney

Williams, Faustine

Williams, Renee

Wilson, Brooke E.

Wilson, Steven

Wimberly, Courtney

Winter, Alexander

Winzenrieth, Renaud

Wischhusen, Jörg

Witzig, Thomas

Woehrer, Adelheid

Wong, Kah Keng

Wong, Ping Pui

Wong, Shun

Woods, Laura

Woolbright, Benjamin

Worni, Mathias

Wösten ‐ van Asperen, Roelie

Wright, Jonathan

Wright, Marilyn

Wu, Alex

Wu, Chen‐Jiang

Wu, Dongmei

Wu, Erxi

Wu, Gaosong

Wu, Hao

Wu, Jiayuan

Wu, Jie

Wu, Jing‐Xun

Wu, Kongming

Wu, Qi‐Jun

Wu, Shengde

Wu, Siqi

Wu, Tsu‐Yin

Wu, Weizhu

Wu, Xiang‐Guang

Wu, Xian‐Ning

Wu, Xiaohua

Wu, Xiaojian

Wu, Xizeng

Wu, Xuan

Wu, Yishuo

Wu, Yufeng

Wu, Ze‐Shen

Wu, Zhenyu

Wurdak, Heiko

Xi, Tao

Xia, Jinglin

Xia, Li‐Qun

Xia, Zhong‐jun

Xiang, Michael

Xiang, Xiaojun

Xiao, Guodong

Xiao, Xiu‐ying

Xiao, Yi

Xiao, Zhiyu

Xie, Conghua

Xie, Qi‐qi

Xie, Shu‐Yang

Xie, Xiao‐ming

Xing, Hui

Xing, Xie

Xiong, Momiao

Xu, Ben‐Hua

Xu, Bing

Xu, Chunwei

Xu, Donghua

Xu, Jing

Xu, Kailin

Xu, Lei

Xu, Midie

Xu, Ming

Xu, Qiuran

Xu, Ran

XU, Song

Xu, Wang‐Hong

Xu, Wei

Xu, Xiao

Xu, Xin‐Jian

Xu, Xiping

Xu, Yi

Xu, Ying‐ying

Xu, You

Xu, Yuexin

Xu, Yuqing

Xu, Zheng

Xu, Zhengshui

Xuan, Yanhua

Yamada, Tadaaki

Yamanaka, Ryuya

Yamanaka, Takeharu

Yan, Caiwang

Yan, Jieyu

Yan, Lei

Yan, Li

Yan, Shushan

Yan, Yongcong

Yan, Zhiling

Yanagi, Teruki

Yang, Xiaotang

Yang, Burton B.

Yang, Dawei

Yang, Grace Meijuan

Yang, Guang

Yang, Hong

Yang, Huijun

Yang, James C.

Yang, Keming

Yang, Lei

Yang, Lihua

Yang, Liuting

Yang, Min

Yang, Ming

Yang, Sen

Yang, Si

Yang, Tian

Yang, Wanshui

Yang, Wei

Yang, Xinrong

Yang, Zhenyu

Yang, Zhibin

Yao, Bowen

Yao, Haijun

Yao, Hongyan

Yao, Ji‐Jin

Yao, Jinjuan

Yao, Min

Yao, Yi‐Chen

Yao, Yue

Ye, Feng

Ye, Jian

Ye, Zeng Jie

Ye, Zhaoming

Yeh, Norman

Yeong, Joe

Yi, Wen‐jun

Yi‐Frazier, Joyce

Yilmaz, Deniz

Yin, Kai

Yin, Ming

Yin, Yongmei

Ying, Hou‐Qun

Ylosmaki , Erkko

Yomo, Shoji

Yoon, Sung‐Soo

Yoshimura, Ryoichi

Yoshizumi, Tomoharu

Young, Kelvin

Yu, Aiping

Yu, Anthony

Yu, Cheng‐Chia

Yu, Chengjun

Yu, Evan

Yu, Hongping

Yu, Hsiang‐Hsuan

Yu, James

Yu, Keda

Yu, Ke‐Da

Yu, Lin

Yu, Qiangfeng

Yu, Shun

Yu, Weiwei

Yu, Xianjun

Yu, Xue Qin

Yu, Zheng

Yu, Zhi Gang

Yuan, Ji‐hang

Yuan, Shuang‐Hu

Yuan, Song‐Hua

Yuan, Xianglin

Yuan, Ye

Yuan, Ying

Yuan, Yuan

Yun, Jae Won

Yun, Sook Jung

Yung, Alfred

Yusoff, Abdul Aziz Mohamed

Zahnd, Whitney

Zaitsu, Masayoshi

Zambello, Renato

Zaucha, Jan Maciej

Zdravkovic, Marko

Zeidan, Amer M

Zeng, Hao

Zeng, Hongmei

Zeng, Jiang‐hui

Zeng, Song

Zeng, Xian‐tao

Zeng, Zhili

Zeng, Zhu

Zeng, Zhuoying

Zer, Alona

Zhan, Cheng

Zhan, Jun

Zhan, Qian

Zhang, Xudong

Zhang, Yong‐Gang

Zhang, Ben

Zhang, Bin

Zhang, Chuan‐Hua

Zhang, Chunlong

Zhang, Dongyu

Zhang, Erbao

Zhang, Hanbin

Zhang, Hao

Zhang, Honghe

Zhang, Hua

Zhang, Hui Lai

Zhang, Jian

Zhang, Jie

Zhang, Jilei

Zhang, Jing

Zhang, Jinmei

Zhang, Jinqiang

Zhang, Jun

Zhang, Kai

Zhang, Li

Zhang, Li‐jin

Zhang, Li‐li

Zhang, Lin

Zhang, Mingran

Zhang, Peng

Zhang, Ruoxin

Zhang, Shichuan

Zhang, Shuijun

Zhang, Shuisheng

Zhang, Shuyu

Zhang, Tingyou

Zhang, Wanfeng

Zhang, Wang‐Jian

Zhang, Wei‐Na

Zhang, Xia

Zhang, Xiaoyu

Zhang, Xipeng

Zhang, Xu

Zhang, Yi

Zhang, Yimu

Zhang, Yiyin

Zhang, Yubao

Zhang, Zeng

Zhang, Zhan

Zhang, Zhigang

Zhao, Bin

Zhao, Haifeng

Zhao, Haitao

Zhao, Helen

Zhao, Jingxuan

Zhao, Lin

Zhao, Ming

Zhao, Peng

Zhao, Shaojie

Zhao, Shujun

Zhao, Wei

Zhao, Yang

Zhao, Yue

Zhao, Zhiqing

Zhen, Jianzhuo

Zheng, Mingjun

Zheng, Shu‐Sen

Zhong, Xiaowu

Zhou, Caicun

Zhou, Can

Zhou, Dong

Zhou, Jiajia

Zhou, Jian‐Guo

Zhou, Jifang

Zhou, Jun

Zhou, Leyuan

Zhou, Meng

Zhou, Qing

Zhou, Quan

Zhou, Wenbin

Zhou, Zhiaing

Zhou, Zhihua

Zhu, Chen

Zhu, Chuanwu

Zhu, Guoxing

Zhu, Hong‐Hu

Zhu, Hui

Zhu, Ji

Zhu, Jie

Zhu, Jinhong

Zhu, Liancheng

Zhu, Meng

Zhu, Ruiqi

Zhu, Weian

Zhu, Xiao

Zhu, Xiaodong

Zhu, Yi

Zhuang, Guohong

Zhukova, Nataliya

Zoccarato, Marco

Zokaasadi, Mohammad

